# Development of a novel colorectal endoscopic submucosal dissection training model

**DOI:** 10.1055/a-2094-8897

**Published:** 2023-06-15

**Authors:** Tomohiro Mitsui, Hironori Sunakawa, Seiichiro Takayama, Tatsuro Murano, Kensuke Shinmura, Hiroaki Ikematsu, Tomonori Yano

**Affiliations:** 1 Department of Gastroenterology and Endoscopy, National Cancer Center Hospital East, Kashiwa, Japan; 2 Division of Endoscopy, Saitama Cancer Center, Saitama, Japan; 3 NEXT Medical Device Innovation Center, National Cancer Center Hospital East, Kashiwa, Japan; 4 KOTOBUKI Medical, Inc., Saitama, Japan; 5 Division of Science and Technology for Endoscopy, Exploratory Oncology Research & Clinical Trial Center, National Cancer Center, Kashiwa, Chiba, Japan

Colorectal endoscopic submucosal dissection (ESD) requires technically advanced endoscopic skill. To improve endoscopic skills, animals or their extracted digestive tracts are used for ESD training 1 2 3 , which raises several issues regarding ethics, cost, and infection.

Applying our previously developed gastric ESD model 4 , we have developed a colorectal ESD training model using non-animal materials with KOTOBUKI Medical, Inc. (Saitama, Japan) to resolve these issues ( Fig. 1 ). This plant-derived model is superior to traditional animal models because training can occur at any time in an endoscopy room, using personal endoscopes and devices, without concern over infection risk. These advantages allow training to be performed multiple times, promoting muscle memory, which is essential for improving ESD skills 5 . The model modified for colorectal ESD allows for training on lesions at any wall location, basic scope movement, necessary strategies for colorectal ESD, changes in air volume in the colon, changes in gravity due to body posture changes, and scope movement with a retroflexed view ( Fig. 2 ).

**Fig. 1:**
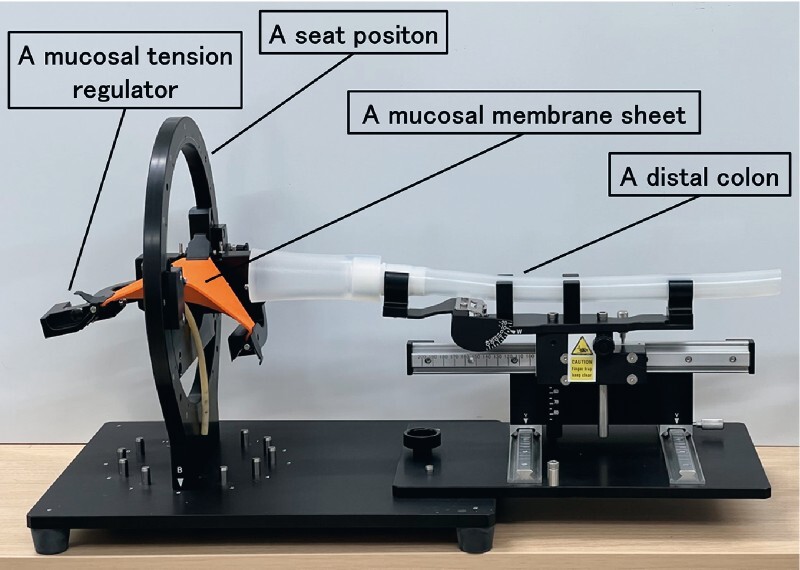
The model consists of a seat position with a two-axis gimbal structure, mucosal tension regulator that can reproduce the change in air volume in the colon during endoscopic submucosal dissection and adjust the tension on demand, mucosal membrane sheet, and distal colon.

**Fig. 2:**
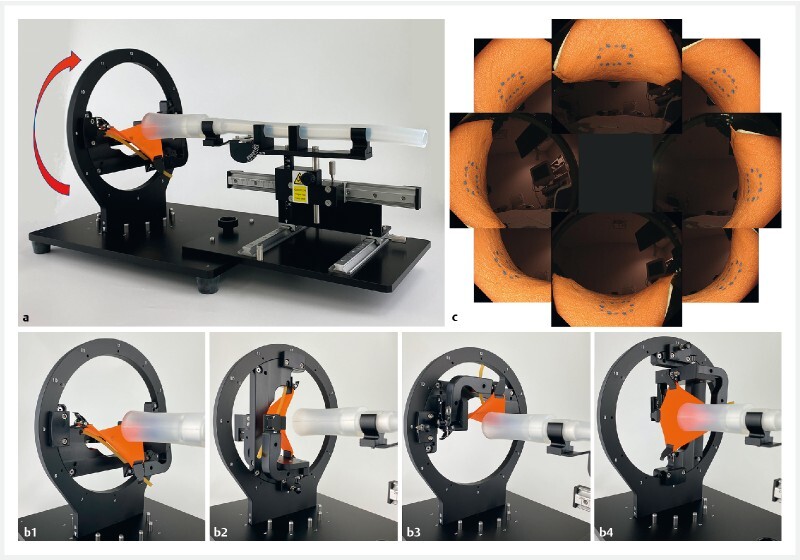
Use of the model in training. **a** The model allows colonic endoscopic submucosal dissection of lesions at any wall location by revolving the setting frame. **b-1** Lesion below the lumen. **b-2** Lesion to the left of the lumen. **b-3** Lesion above the lumen. **b-4** Lesion to the right of the lumen. **c** Endoscopic imaging of the lesion at any wall location.

In this study, the modified model was used and a specialized liquid (VTT-INJ; KOTOBUKI Medical, Inc.) was injected into the submucosal layer. Mucosal circumferential incision and trimming, and submucosal dissection were performed using a dual knife ( Video 1 ). After ESD, a questionnaire about the similarities in clinical practice between the conventional and new colorectal ESD model, and the usefulness of the new model for improving trainees’ ESD skills was answered on a six-point scale (0–5) ( Fig. 3 ). Three colorectal ESD experts who were not involved in developing this training model and who had performed more than 100 clinical colorectal ESDs completed the questionnaire.

**Video 1** Colonic endoscopic submucosal dissection was performed on this model.


**Fig. 3:**
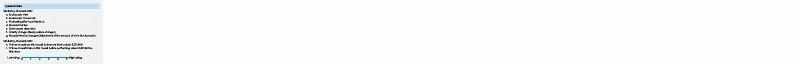
The questionnaire responses were scored on a six-point scale (0–5). Participants subjectively scored for similarity to conventional colonic endoscopic submucosal dissection (ESD) regarding a) endoscopic view, b) endoscopic movement, c) protrusion after local injection, d) mucosal incision, e) submucosal dissection, f) gravity changes due to body posture changes, and g) mucosal tension changes, reproducing the change in air volume in the colon during ESD, and adjusting the sheet for the tension or to loosen it on demand. Participants also scored for whether trainees should use this model to h) improve their colonic ESD skills and i) train before performing colonic ESD for the first time.

All responses for the similarities between the new model and conventional colorectal ESD ( Fig. 4 a ) and the acceptability of the new model ( Fig. 4 b ) scored ≥ 4 and 5 points, respectively. ESD training using the novel model is similar to the sensation of clinical colorectal ESD and is useful for trainees.

**Fig. 4:**
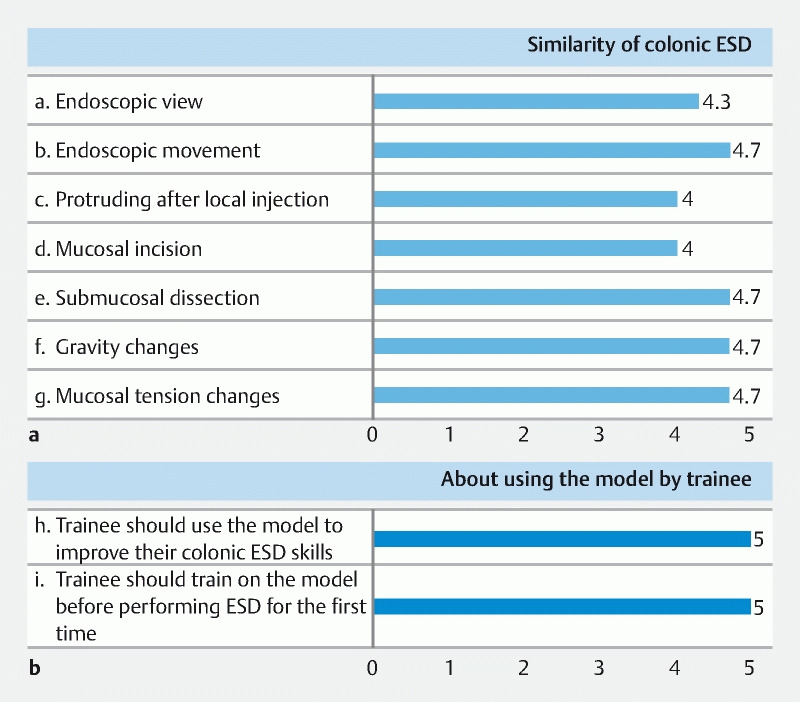
Questionnaire responses. **a** The question about the similarity to conventional colonic endoscopic submucosal dissection scored 4 points or higher. **b** The question about whether trainees should use this model scored 5 points.

Endoscopy_UCTN_Code_TTT_1AQ_2AD

## Notes

Endoscopy E-Videos

https://eref.thieme.de/e-videos
*E-Videos* is an open access online section of the journal *Endoscopy* , reporting on interesting cases and new techniques in gastroenterological endoscopy. All papers include a high-quality video and are published with a Creative Commons CC-BY license. Endoscopy E-Videos qualify for HINARI discounts and waivers and eligibility is automatically checked during the submission process. We grant 100% waivers to articles whose corresponding authors are based in Group A countries and 50% waivers to those who are based in Group B countries as classified by Research4Life (see: https://www.research4life.org/access/eligibility/ ). This section has its own submission website at https://mc.manuscriptcentral.com/e-videos
